# Population structure and genetic diversity of mango (*Mangifera indica* L.) germplasm resources as revealed by single-nucleotide polymorphism markers

**DOI:** 10.3389/fpls.2024.1328126

**Published:** 2024-07-03

**Authors:** Qingzhi Liang, Hongbing Pan, Xiaolong He, Songbiao Wang, Yuanhua Hou, Hua Xiao, Guangzhao Xu, Runhua Yi, Dongbo Lin, Zhuanying Yang

**Affiliations:** ^1^ College of Coastal Agricultural Sciences, Guangdong Ocean University, Zhanjiang, China; ^2^ South Subtropical Crops Research Institute, Chinese Academy of Tropical Agricultural Sciences, Zhanjiang, China; ^3^ Fruits Research Institute, Panzhihua Academy of Agricultural and Forestry Sciences, Panzhihua, China; ^4^ College of Tropical Crops, Yunnan Agricultural University, Puer, China; ^5^ College of Electronic and Information Engineering, Guangdong Ocean University, Zhanjiang, China

**Keywords:** *Mangifera indica*, SLAFs, population structure, genetic diversity, SNPs

## Abstract

**Introduction:**

Mango is a vital horticultural fruit crop, and breeding is an essential strategy to enhance ongoing sustainability. Knowledge regarding population structure and genetic diversity in mango germplasm is essential for crop improvement.

**Methods:**

A set of 284 mango accessions from different regions of the world were subjected to high-throughput sequencing and specific-locus amplified fragment (SLAF) library construction to generate genomic single-nucleotide polymorphism (SNP).

**Results:**

After filtering, raw data containing 539.61 M reads were obtained. A total of 505,300 SLAFs were detected, of which, 205,299 were polymorphic. Finally, 29,136 SNPs were employed to dissect the population structure, genetic relationships, and genetic diversity. The 284 mango accessions were divided into two major groups: one group consisted mainly of mango accessions from Australia, the United States, Cuba, India, Caribbean, Israel, Pakistan, Guinea, Burma, China, and Sri Lanka, which belonged to the Indian type (P1); the other group contained mango accessions from the Philippines, Thailand, Indonesia, Vietnam, Cambodia, Malaysia, and Singapore, which belonged to Southeast Asian type (P2). Genetic diversity, principal component analysis (PCA), and population structure analyses revealed distinct accession clusters. Current results indicated that the proposed hybridization occurred widely between P1 and P2.

**Discussion:**

Most of the accessions (80.99%) were of mixed ancestry, perhaps including multiple hybridization events and regional selection, which merits further investigation.

## Introduction

Mango (*Mangifera indica* L., 2n = 40) is an important tropical and subtropical fruit that belongs to the Anacardiaceae family (Wang et al., 2020; Zhou et al., 2020). Mango, the “king of tropical fruits,” is an economically important and the fifth largest fruit crop in the world. The tropical area Southeast Asia is the main growing and production center for mangoes (Luo et al., 2016; Lora and Hormaza, 2018; Liang et al., 2022). At present, globally more than 100 countries grow mangoes, spanning between 30° north and 30° south latitude. India has the largest area of mango cultivation, which accounts for 40%, followed by China, approximately 17% (Lal et al., 2017; Niu et al., 2021; Liu et al., 2023). Approximately 1,370 years ago, mango cultivation started in China and mango was introduced to Southeast Asia (Mukherjee and Litz, 2009; Gao et al., 2011). Mainly five species of mango were cultivated in China, and approximately 200 cultivars belonging to *Mangifera indica* L. were released for cultivation (Zhou et al., 2020).

Around 69 species of the mango genus, mainly found in India, Sri Lanka, China, and Philippines (Bompard, 2009; Gao et al., 2011; Lora and Hormaza, 2018), one specie (*Mangifera indica*) and more than 1,000 mango cultivars were currently cultivated worldwide (Wang et al., 2020). Botanically, there are two main groups: (i) monoembryonic type, where the seed has only one embryo and only one seedling emerges after sowing, (ii) polyembryonic type: where the seeds have multiple embryos and after sowing several seedlings can grow—mango accessions from Philippine varieties and Thailand varieties belong to this type (Mukherjee and Litz, 2009; Warschefsky and Wettberg, 2019).

Mango had been cultivated in India and Indochina for more than 4,000 years before being introduced to Africa, South America, and other continents (Popenoe, 1920; Mukherjee, 1949; Singh et al., 2016; Warschefsky and Wettberg, 2019). Mangoes arrived in Miami from the West Indies in 1862 or 1863 (Litz, 2009). During the twentieth century, with the implementation of a breeding program in South Florida, more and more elite commercial cultivars were released; some of them are still the dominant varieties in mango-producing regions (Knight et al., 2009). Historically, mango was introduced to China; two possible routes were proposed. The first recorded introduction of mangoes was in the Tang Dynasty (Mukherjee and Litz, 2009; Gao et al., 2011); mango was secondly introduced in China from India and South Asia in the fifteenth century AD by sea during Zheng He’s Voyages to the Western Seas (Ming Dynasty. Xu, 1992; Yang, 2019). The mango breeding program in China had gone through two stages: the first stage was seedling breeding, where a large number of original varieties were selected from seedlings of Indian or Southeast Asian type varieties (P1 or P2); the second one was cross breeding, where the artificial cross breeding between P1 and P2 produced many excellent varieties which had been widely promoted in China (Luo et al., 2010; Huang et al., 2013; 2012).

Mango cultivation has greatly promoted the income of farmers in tropical regions; however, its molecular biology research was relatively lagging behind (Warschefsky and Wettberg, 2019). In recent years, with the development of molecular biology, the development of molecular markers and genetic diversity evaluation of mango germplasm resources have made some progress (Nadeem et al., 2017; Zhou et al., 2020). Previous studies used nuclear DNA sequence fragments such as RAPD (Schnell et al., 1995), AFLP (Yamanaka et al., 2006); ISSR (Singh et al., 2007), SSR (Shamili et al., 2012), SCoT (Zhou et al., 2020), and SNP (Sherman et al., 2015; Kuhn et al., 2019) markers to evaluate the genetic diversity of mango germplasm resources. However, further research on mango biology was limited due to the limited number of polymorphic molecular markers. The development of mango chromosome-level whole genome sequencing (Wang et al., 2020; Ma et al., 2021; Mango Genome Consortium et al., 2021) and high-throughput sequencing technology has made it possible for mango to develop bulk molecular markers quickly and cheaply.

Specific-locus amplified fragment sequencing (SLAF-seq) technology was an efficient method, which was developed by Beijing Biomarker Technologies Corporation (Sun et al., 2013). To date, SLAF-seq has been used successfully to dissect accurately the genetic diversity and population structure for many crops (Shen et al., 2017; Zeng et al., 2017; Chang et al., 2019). The results of such techniques were further used in plant breeding (Jones et al., 2013; Boeven et al., 2016; Wu et al., 2016; Zhang et al., 2018; Bruce et al., 2019; Cui et al., 2020).

In the current study, 284 mango germplasm resources were collected from different regions worldwide. However, the genetic diversity of these mango germplasm resources in the collection remained molecularly uncharacterized. Therefore, this research analyzed the genetic diversity and population structure of 284 mango germplasm resources using SNP markers in order to generate highly important information, which together with previous research results led to deeper insight on the mango gene pool for mango breeders worldwide.

## Materials and methods

### Sampling

In order to dissect the population structure and genetic diversity of mango germplasm resources in China, 284 mango accessions were selected from National Field Genebank for Tropical Fruit (Zhanjiang, China) that originated in different geographical regions: a total of 18 countries or regions of major mango growing areas of the world, mainly including India, Pakistan, Thailand, Burma, Sri Lanka, Indonesia, the Philippines, Australia, the United States, China, etc. These accessions were collected from different geographical areas worldwide since the 1920s (from the earliest introduction of new mango varieties by overseas Chinese to the later introduction and exchange of germplasm resources worldwide); we tried to collect more diverse and distinctive mango germplasm resources in morphology and geography from every tropical region, including historical varieties particularly. Additionally, we collected also landrace, new varieties, and elite lines identified through the Chinese breeding program (**Supplementary Table S1**).

### DNA extractions and SLAF-seq

Genomic DNA from young leaves of 284 mango accessions were extracted using the modified CTAB method described by He et al. (2005). The quality and purity were determined with 1.2% agarose gel electrophoresis and spectrophotometry at a wavelength of 260/280 nm using a BioPhotometer (D30, Eppendorf, Germany), respectively. In the present study, 284 mango germplasm resources were subjected to molecular marker development using the SLAF-seq approach, which was developed by Beijing Biomarker Technologies Corporation to obtain genome-wide molecular markers (Sun et al., 2013). Except that the genomic DNA was digested with double restriction enzymes Hpy166II and EcoRV. Sequences with an enzyme digestion length of 264 bp–414 bp were defined as SLAF tags. These SALF tags were aligned with the latest mango genome sequences (https://www.ncbi.nlm.nih.gov/assembly/GCA_016746415.1) and localized to the corresponding chromosomes. SNPs were obtained using GATK and SAMtools (Li and Durbin, 2009; McKenna et al., 2010). SNPs with minor allele frequency (MAF) < 5% and integrity < 80% were excluded from the genotype data sets of all the accessions.

### Data analysis

The mean effective numbers of observed heterozygosity (Ho), expected heterozygosity (He), and fixation index (Fst) for each SNP marker and accession were estimated using software PLINK v1.90b6.21. Population genetic structure analysis is an important tool for genetic relationship analysis, which can provide information about the origin and composition of individual lineages. Based on 29,136 polymorphic SNP markers, the population structure of 284 germplasm resources was analyzed using STRUCTURE V2.3.4 software (Alexander et al., 2009; Excoffier and Lischer, 2010). To determine the number of hypothetical clusters (K), define populations, and assign individual accessions to certain subpopulations based on genetic data, an admixture and shared allele frequencies model was employed (Pritchard et al., 2000). Numbers in the range from 1 to 10 were assumed for K. The initial burn-in period, for each run, was set to 10,000 with 100,000 MCMC (Markov chain Monte Carlo) iterations, with no previous information on the source of accessions. The most suitable value of K was calculated using the ΔK method as used in the Structure Harvester web page (Evanno et al., 2005).

A phylogenetic tree was used to represent the evolutionary relationship among mango germplasm resources. According to the distance of the relationship between various organisms, all kinds of organisms were placed on the branched tree chart, which showed the evolutionary process and relationship of organisms. Based on SNP markers, the phylogenetic tree of 284 mango accessions was analyzed by RAxML version 8.2.12 software and the neighbor-joining algorithm (Saitou and Nei, 1987; Tamura et al., 2011). A bootstrap consensus tree was obtained from 1,000 replicates.

Principal component analysis (PCA) of the selected SNPs was performed with Plink v1.90b6.21 software. Based on SNP markers and cluster software, principal component analysis (PCA) was carried out to obtain the clustering of 284 mango germplasm resources. PCA can be used to determine whose samples were relatively close or whose samples were relatively distant, which can assist evolutionary analysis (Dunteman, 1989; de Hoon et al., 2004). Meanwhile, to explore the similarities and dissimilarities among samples, principal coordinate analysis (PCoA) was also conducted. A distance matrix was calculated using Euclidean distance from the PCA matrix. PCoA was then performed using the cmdscale function in R, retaining two dimensions (k = 2). The resulting coordinates were transformed into a data frame, and sample groups were assigned for visualization purposes. Regions of interest were identified based on the data distribution, with specific coordinates highlighting dissimilar and similar areas. Visualization of the PCoA results was achieved using the ggplot2 package, incorporating enhanced point size, transparency (alpha = 0.6), and a minimal theme. Text size and font were adjusted, and colors were manually set for different groups. Rectangular annotations were used to highlight the identified regions of interest.

## Results

### SLAF-seq genotyping

Using the SLAF-seq approach developed recently, a total of 539.61-M paired-end reads for the 284 mango accessions were obtained. For the reads, on average, Q30 was 88.94% and the GC content was 36.12% (**Supplementary Table S2**). High-quality reads were aligned to the reference genome of mango (https://www.ncbi.nlm.nih.gov/assembly/GCA_016746415.1).

**Figure 1:**
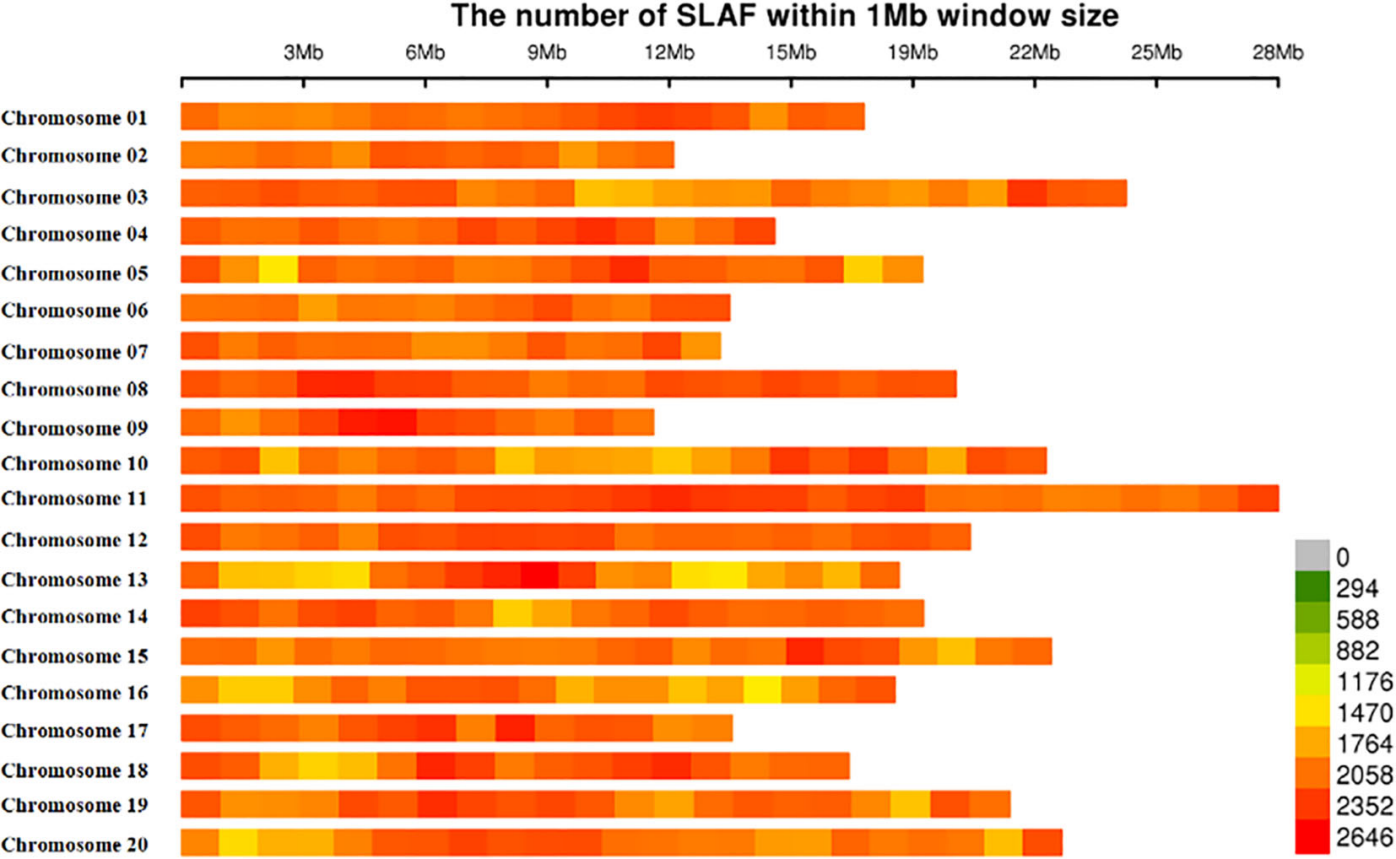
Chromosomal distribution of 778, 000-SLAFs used in this study. Single-nucleotide polymorphism (SNP) distributions on all 20 chromosomes of mango. The horizontal axis shows chromosome length; the 0–2646 values in the legend inset depict SNP density.

The number of reads for each mango accession was not equal, which ranged from 1,058,866 to 11,753,604, with an average of 1,900,029. After read clustering, 505,300 SLAF markers were detected and the average sequencing depth was 9.98X. Of the 505,300 SLAF markers, 205,299 SLAF markers were polymorphic, with the polymorphism rate reaching up to 40.63%, and the remaining 300,001 SLAF markers were non-polymorphic or repetitive. A total of 778,000 SLAFs were successfully invoked to evaluate the MAF and integrity based on accessions, which were distributed evenly throughout the mango genome (**Supplementary Table S2**; **Figure 1**).

Those 778,000 SLAF markers evenly distributed across all 20 chromosomes, and the number of SLAFs on each chromosome corresponds to its physical length (**Figure 1**; **Supplementary Table S4**). The largest number of SLAFs was detected on chromosome 11 (61,172 SLAFs), followed by chromosome 3 (48,775 SLAFs), whereas the smallest number of SLAFs was found on chromosome 9 (26,173 SLAFs).

Observed heterozygosity (Ho) varied from 0.14 to 0.33 with a mean of 0.22 (**Supplementary Table S3**), whereas the mean expected heterozygosity (He) was 0.11 with individual values per locus ranging from 0.07 to 0.16 (**Supplementary Table S3**). In the case of fixation indices, the minimum, maximum, and mean values for F_ST_ were 0.03, 0.72, and 0.47, respectively (**Supplementary Table S3**; **Supplementary Figure S1**).

### Population structure and admixture analysis

**Figure 2:**
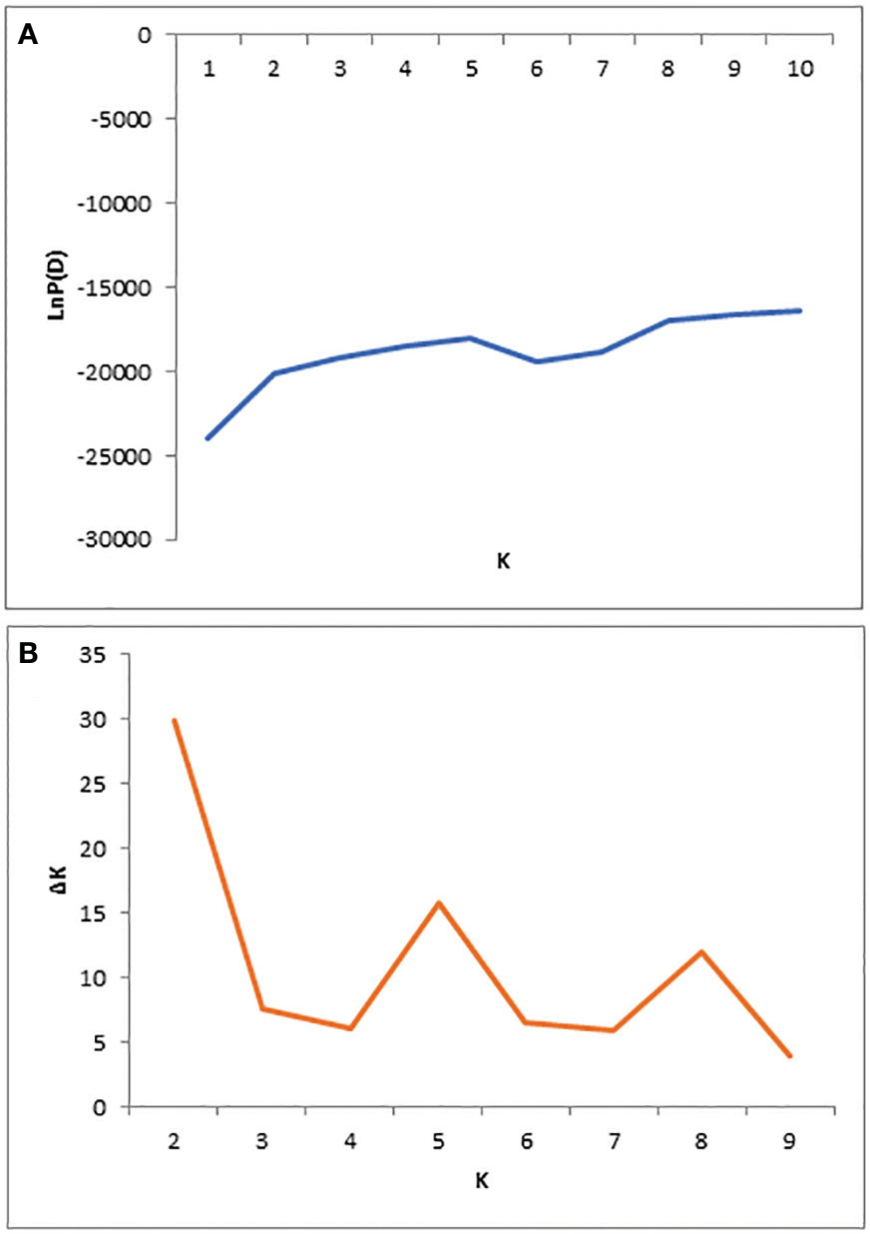
Population structure of 284 diverse genotypes; graphical presentation of the estimation of posterior probability LnP(D) **(A)** and ΔK **(B)**.

**Figure 3:**
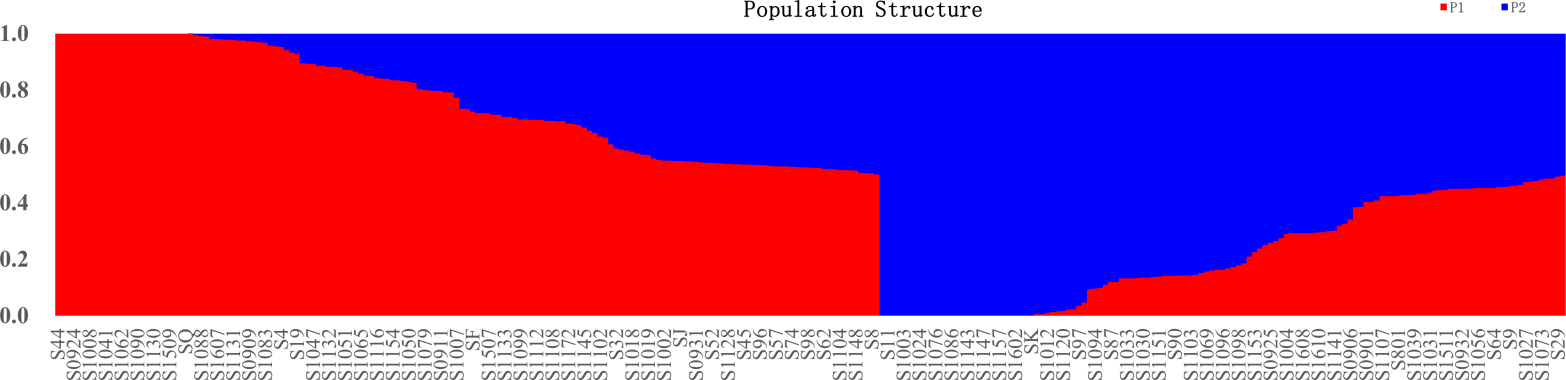
Population STRUCTURE analysis of 284 mango accessions using 29,136 SNP markers. The proportion of membership in each group and subgroups at ΔK = 2 as defined with a model-based clustering method from Pritchard et al. (2000) based on SNP markers. The individual sample was coded in a way that the first letter (S) plus germplasm number represented the names of mango accessions name. Red represents Indian-type accessions (P1); blue represents Southeast Asian-type accessions (P2).

The population structure of 284 mango germplasms was evaluated with 29,136 SNP markers using STRUCTURE V2.3.4 software (**Figures 2**, **3**). The LnP(D) score for the number of populations (K) is shown in **Figure 2A**; no significant inflation point was found. The peak of ΔK occurred when K was 2 (**Figure 2B**). According to the principle of maximum likelihood value, combined with the method of K value determination by Evanno et al. (2005), the best K value was judged to be equal to two; therefore, 284 mango germplasm resources were classified into two subpopulations (**Figures 2**, **3**).

To understand the origin and breeding history of mango, ancestry proportions of 284 mango accessions were estimated by the Bayesian clustering algorithm. In the present study, 284 mango accessions were classified into two subpopulations based on Q (the probability that the genomic variation of a material A originates from population K) less than or equal to 0.5 (**Figure 3**; **Supplementary Table S5**). Of 284 accessions, 155 (54.58%) accessions were assigned to P1 and the remaining 129 (45.42%) accessions were assigned to P2. These results showed that the mango accessions analyzed, regardless of their current distribution, had ancestors in both populations. Admixture analysis identified 284 accessions in the attribution and showed that the gene flow of cross-pollination derived from a mixed ancestral origin (**Supplementary Table S3**; **Figure 3**). Of those, 25 accessions of P1 kept their homogeneous genetic background; these accessions were either introduced directly from India historically or hybrids of Indian varieties (**Figure 3**; **Supplementary Table S1**). Similarly, 29 accessions with a homogeneous genetic background come from P2; these accessions were either introduced directly from India-China historically or hybrids of Indian-Chinese varieties (**Figure 3**; **Supplementary Table S5**). In addition, 130 and 100 cultivars had a mixed pedigree belonging to P1 and P2, respectively (**Figure 3**; **Supplementary Table S5**). The recent introgression from P1 into P2 indicated that some of different populations did not reflect their geographical distributions.

For K equaling two, population 1 mainly from Florida, India, Australia, Sri Lanka, Cuba, Israel, and the Caribbean had a similar composition, with a high-level ancestry of P1 and a moderate admixture level ancestry of P2. The majority of accessions in Indonesia and Southeast Asia (Philippines, Thailand, Cambodia, Malaysia, Singapore, and Vietnam) had the highest level ancestry of P2 and a moderate admixture level ancestry of P1. In addition, there were 108 mango accessions from China, 59 of whose accessions had high-level ancestry from P1, and the remaining 49 mango accessions had high-level ancestry from P2, with great variability in inferred ancestry across mango accessions; these results showed most of mango accessions from China with a high level of admixture from the two populations, indicative of the ongoing exchange of mango germplasm resources around the world.

**Figure 4:**
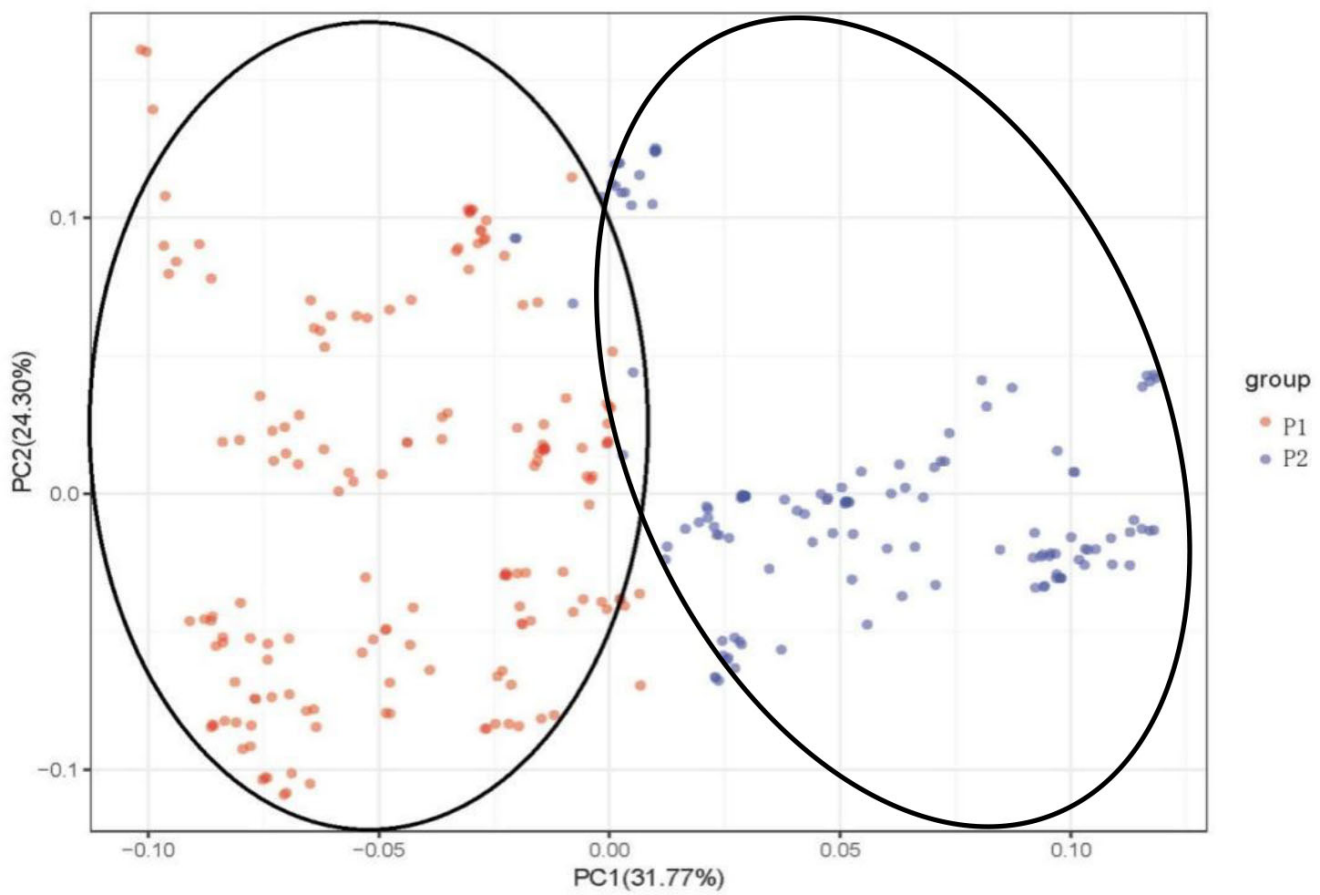
Principal component analysis (PCA) of 284 mango germplasm resources collected from different tropical regions worldwide. The percentage of variation explained by the corresponding PC is marked on the axis. Red represents Indian-type accessions (P1); blue represents Southeast Asian-type accessions (P2).

Principal component analysis (PCA) was used to analyze the attribution of 284 mango accessions to better understand the relationship among 284 mango germplasm resources, and PCA scores were used to assess genetic variation (**Figure 4**). The first principal component (PC1) explained 31.77% of the variance, whereas the second (PC2) explained 24.30% (**Figure 4**). According to the first two components, the accessions were divided into two groups: Indian type (P1) and Southeast Asian type (P2). Once again, the results of the PCA were consistent with the results of the population structure. The two groups can still be clearly distinguished, although a few accessions overlap between P1 and P2, following a trajectory suggestive of admixture between P1 and P2.

Comparison by horticultural type (monoembryonic vs. polyembryonic) was characterized for the 284 germplasm resources (**Supplementary Table S1**). Both clusters showed a mixture of polyembryonic and monoembryonic seed-type varieties in their cluster. However, most germplasm resources (99 accessions) in P1 were single-embryonic seeds, whereas majority of germplasm resources (99 accessions) in P2 were polyembryonic seeds (**Supplementary Table S1**), which is consistent with the characteristics of the Indian mango type and the Indochinese mango type, respectively. These results indicated that mango (*Mangifera indica* L.) had two places of origins: one was India, and the other was Indochina Peninsula. From the embryonic point of view, mango cultivars of the United States and Australia belonged mostly to the Indian type (P1). Both populations showed a mixture of polyembryonic and monoembryonic seed-type varieties in their cluster.

### Genetic diversity and genealogy analysis

**Figure 5:**
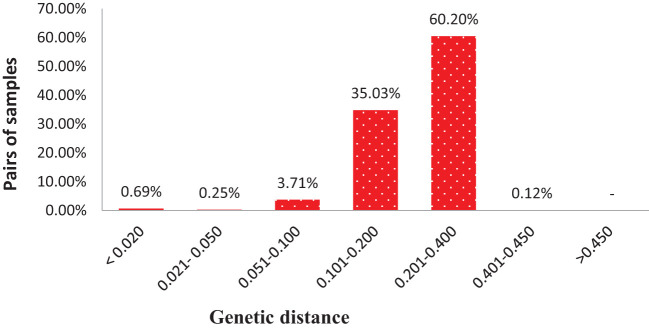
Distribution of pairwise identity-by-state based genetic distances among pairs of 284 mango accessions.

DARwin 6 software was used to calculate the genetic distance coefficients between collected mango accessions based on the proportions of shared alleles obtained from SNP markers for each accession. Genetic distances between pairs of the 284 accessions varied from 0.002 to 0.450 (on a scale of 0–1, 0 means no parentage relationship at all and 1 means the same accession), with an overall average of 0.207 (**Supplementary Table S6**). The 39,798 pairwise comparisons are summarized in **Figure 5**, where most pairs had distances ranging from 0.100 to 0.400; however, 372 pairs of genotypes (0.93%) had genetic distance ≤0.05 and 273 pairs differed by ≤2% of the total number of alleles in present analyses. Majority of pairs of genotypes (95.24%) had a genetic distance between 0.101 and 0.400. No pairs of accessions had a genetic distance over 0.500; these results suggested 284 accessions with high levels of diversity (**Figure 5**; **Supplementary Table S6**).

**Figure 6:**
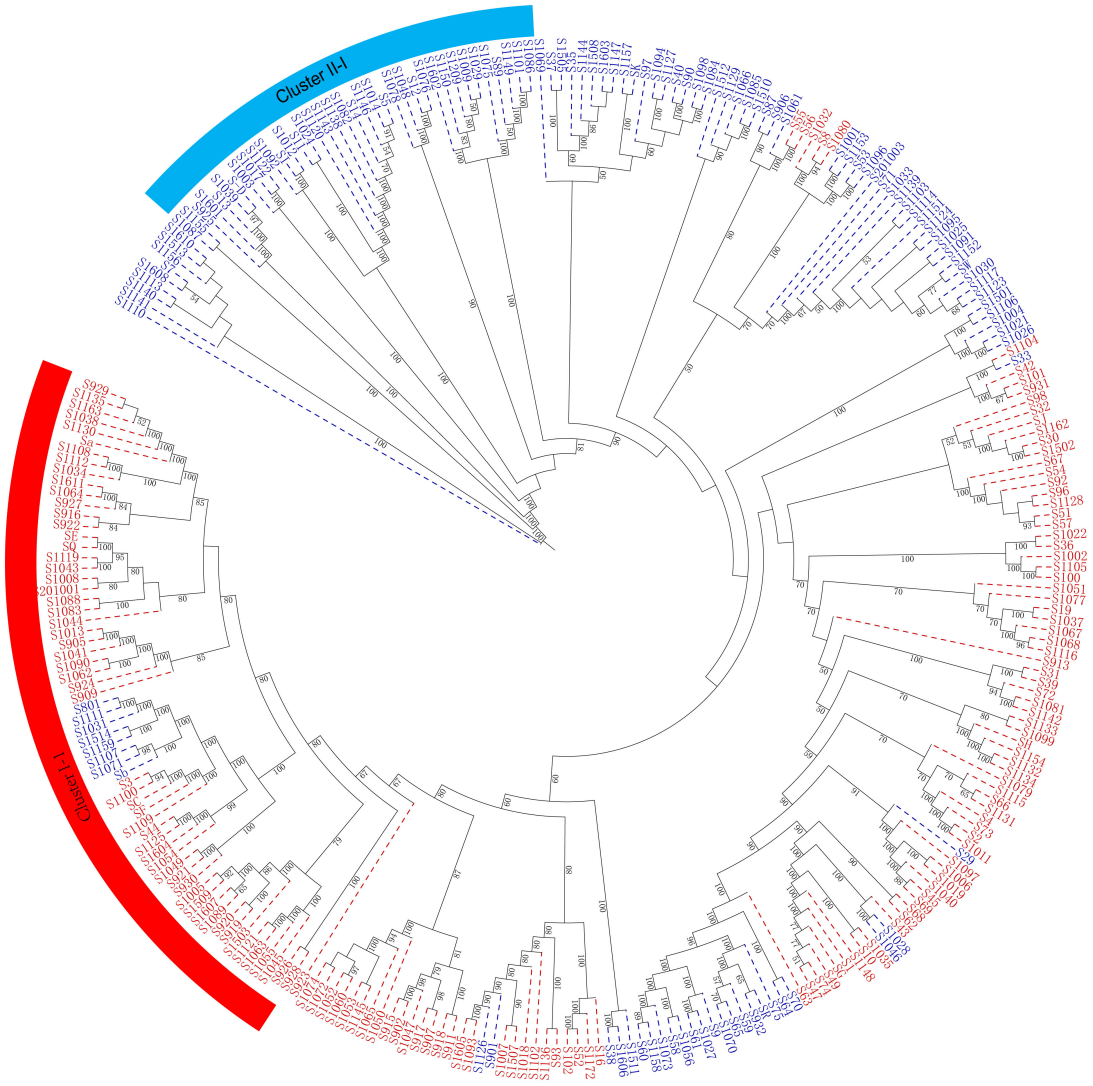
A maximum likelihood phylogenetic tree constructed with RAxML software for the 284 mango accessions using the filtered SNPs. The number alongside each node represents the bootstrap values. The individual sample was coded in a way that the first letter (S) plus germplasm number represented the names of mango accessions name. Red represents Indian-type accessions (P1); blue represents Southeast Asian-type accessions (P2).

The genetic diversity and genetic relationship of 284 mango accessions were further elucidated using the Nei’s genetic distance-based unweighted group averaging cluster analysis method (UPGMA). The present results indicated that 284 mango accessions were divided into two distinct clusters, mainly based on their geographical origin (**Figure 6**).

Cluster I included 141 mango accessions (C1), majority of which originated from regions and countries such as Florida, Australia, India, Cuba, Sri Lanka, Caribbean, and Israel (P1) (**Supplementary Table S1**; **Figure 6**). Notably, of 42 American mango accessions, 39 mango accessions were grouped into cluster I; similarly, 16 of 19 Indian mango accessions and 7 of 9 Australian mango accessions were grouped into cluster I, respectively. Meanwhile, cluster I contained 49 mango accessions from China. Cluster II contained 143 mango accessions (C2) originating mainly from Thailand, Burma, Cambodia, the Philippines, and Indonesia (P2), which also included 59 mango accessions from China. These results indicated that compared with the mango germplasm resources of Southeast Asia, American mango varieties had a closer genetic relationship with Indian mango varieties, and mango germplasm resources in China originated from the Indian type and Southeast Asian type; simultaneously, these results were consistent with the road map of mango’s introduction into China in history.

As expected, it can be seen from the dendrogram that the genetic relationship between derived varieties/landraces and their parent varieties was closely similar. There were 25 of 39 American accessions grouped into cluster I-1 (**Supplementary Table S1**; **Figure 6**), of which 13 accessions resulted from seedlings of Haden or derived from Haden hybrids, the remaining accessions came from hybrids of early mango introduction in America such as ‘Mulgoba,’ ‘Turpentine,’ ‘Sandesina,’ and ‘Bombay’. Population structure analysis showed that these American mango varieties had a mixed and higher proportion ancestry of Indian mango type (P1) (**Supplementary Table S5**). Given that 13 accessions had a much broader genetic base, and the ancestors of the world’s most popular commercial varieties (Olano et al., 2005; Singh et al., 2016), these 13 accessions possessed very unique genes involved with a wide range of adaptability and production stability in growing regions around the world (Mukherjee and Litz, 2009). For this reason, we might refer that the American varieties in cluster I-1 constituted a heterotic group (H1). Exceptionally, only a mango accession ‘Edward’ was grouped into cluster II (Southeast Asian type, P2); pedigree analysis indicated that ‘Edward’ derived from ‘Haden’× ‘Carabao’, a hybrid between P1 and P2 selected under south Florida conditions. Population structure analysis also showed that ‘Edward’ had a mixed ancestry, 54.6% Southeast Asian-type ancestry (P2) and 45.4% Indian-type ancestry (P1). The results indicated that ‘Edward’ was on the borderline of the genetic population between cluster I and cluster II. Meanwhile, another heterotic group was found in cluster II and 25 of 39 Thailand mango accessions were grouped into cluster II-1; a large number (N = 21) of new mango varieties had been selected using seedling breeding from those of Thailand mango germplasm resources and promoted in China. In particular, a series of varieties ‘Guire No. 3,’ ‘Guire No. 7,’ ‘Guire No. 10,’ ‘Yuanjiang_ivory,’ ‘Haibao 1,’ ‘Guire 71,’ ‘Hongxiangya,’ ‘Jinsui,’ ‘Wugongci Mango,’ ‘Guire 11,’ and ‘Hwagnyu (503)’ were selected from Thailand mango accessions and were released and promoted in China (**Supplementary Table S1**); from this perspective, Thailand mango germplasms probably constituted another heterotic group (H2). Interestingly, several important commercial mango varieties were bred by Chinese mango breeders through cross breeding between American (H1) and Thailand varieties (H2); these commercial mango varieties included ‘Jinhuang,’ ‘Yiwen,’ ‘Guixiang,’ and ‘Guifei’; those were evenly distributed in the phylogenetic tree (**Figure 6**; **Supplementary Table S1**).

## Discussion

### Development of high-throughput sequencing markers

The development of abundant and reliable molecular markers was extremely important for mango germplasm resource evaluation and breeding. In the present study, 29,136 SNP markers were used to dissect the population structure, principal component analyses (PCA), and genetic diversity for 284 mango accessions. SLAF-seq technology was a newly developed technique to identify SNP markers in recent years (Kashkush et al., 2001; Sun et al., 2013). Since the development of SLAF-seq technology, it has been applied in many plants studies and has achieved remarkable achievements. Shen et al. (2017) employed SLAF-seq to identify a large number of SLAF markers. Interspecific variation was identified using these markers, and these results were useful for cotton genetics research and molecular breeding. Yang et al. (2020) applied SLAF-seq to develop 8,738 polymorphic SLAFs and resistance genes of soybean mosaic virus strain SC9 identified from cultivar Tianlong No. 1. Approximately 148 target genes were located on chromosome 2. Similar studies were carried out in many plants, such as Japanese plum (Zhang et al., 2020), Zicaitai (Li et al., 2019), eggplant (Wei et al., 2020), Chinese Lou onion (Fang et al., 2020), and Chinese elm (Lyu et al., 2020). Compared with traditional methods, this kind of marker developed by SLAF-seq technology has the advantages of higher density, consistency and effectiveness, and lower cost. In previous studies, RAPD (Schnell et al., 1995; Rajwana et al., 2008), AFLP (Yamanaka et al., 2006), ISSR (Singh et al., 2007), SSR (Shamili et al., 2012), SCoT (Zhou et al., 2020) had been used to dissect the genetic diversity of mango. However, compared with genome-level sequencing, previous molecular markers developed using the traditional method had a lower accuracy and resolution, as well as limited number of polymorphic loci, which hindered genetic research on mango. Recently, Kuhn et al. (2019) used SNP markers to genotype 1,915 mango accessions and estimate genetic diversity and relatedness; however, only 272 SNP markers were polymorphic and the number of markers was relatively small and could not cover the whole genome, which resulted in relatively large average distances between the adjacent markers (Sherman et al., 2015). Enough unbiased SNP assessments would accurately reflect the genome-wide diversity that occurred in natural populations (Zhang et al., 2020).

In the present study, 539.61 M reads were obtained for analysis of the genetic diversity of 284 mango germplasm resources. A total of 1,272,446 SLAFs were detected, of which 156,368 were polymorphic. Finally, 29,136 SLAFs were employed to dissect population structure, principal component analyses (PCA), and genetic diversity. There were more polymorphism markers in mango developed through SLAF-seq technology than the traditional marker technology, which further suggested the potential of SLAF-seq as a low-cost technology to effectively develop a large number of reliable molecular markers in mango.

### Genetic diversity and population structure of mango

The importance of understanding the genetic diversity of mango accessions in various tropical regions worldwide is critical for conservation and utilization of mango germplasm resources and assisting breeders to attain elite varieties in the mango breeding programs.

Although genetic diversity of mango germplasm resources had been previously reported using different types of markers (Schnell et al., 1995; Yamanaka et al., 2006; Singh et al., 2007; Rajwana et al., 2008; Shamili et al., 2012; Zhou et al., 2020), Schnell et al. (1995) suggested that a single RAPD marker cannot distinguish all materials and a combination of two or more markers can effectively distinguish different materials. Yamanaka et al. (2006) dissected four *Mangifera* species using AFLP markers; their results showed that AFLP markers grouped 35 materials into four subgroups, which could effectively distinguish four mango species, and four subgroups were consistent with four species. AFLP markers also clearly revealed the genetic diversity between and within intraspecific and interspecific hybrids of mango. Samal et al. (2012) analyzed the genetic diversity of Indian mango using RAPD and ISSR markers; the results showed that 65 mango germplasm resources were clustered into eight groups, consistent with their pedigree relationships. Shamili et al. (2012) studied the genetic diversity of 41 Iranian mango germplasm resources using 16 SSR markers; cluster analysis showed that Iranian mango originated from India and Pakistan. Zhou et al. (2020) suggested that 168 mango germplasm resources were grouped into two major clusters using SCoT markers; the genetic diversity within populations was much higher than that between populations. There were 34 germplasm resources identified, most of which gathered with their parents. These results indicated that SCoT markers were useful for identification and genetic diversity analyses of mango germplasms. However, previous studies were conducted with only a few markers and limited mango accessions, too few to sufficiently elucidate the genetic diversity of mango accessions. Genetic diversity analysis was a key step in the discovery of alleles that can be used as a source of excellent traits such as high-yielding, resistant to abiotic or biotic stress (Alemu et al., 2020).

The Bayesian model-based structure analysis revealed the presence of two populations among 284 mango accessions. Of the 284 mango accessions analyzed, 155 (54.58%) accessions were assigned to P1 and the remaining 129 (45.42%) accessions were assigned to P2. These results indicated that the two populations of mango analyzed, regardless of their current geographical distribution, had ancestors in mutual populations, these results were similar to that of the phylogenetic tree and PCA analyses; meanwhile, PCoA (**Supplementary Figure S2**) was also performed and the result was consistent with those of PCA. Our result was further consistent with the two domestication centers that have been proposed for a long time: one was in India, and the other was in Southeast Asia (BJaS, 1997; Schnell et al., 2006; Wang et al., 2020). Admixture analysis identified 284 mango accessions showing gene flow from cross-pollination of mixed ancestral origin (**Supplementary Table S5**; **Figure 3**). Of which, 25 mango accessions of P1 kept their homogeneous genetic background; these accessions were either introduced directly from India historically, or hybrids of Indian varieties (**Figure 4**; **Supplementary Table S4**). Similarly, there were 29 mango accessions with a homogeneous genetic background coming from P2; these accessions were either introduced directly from India-China historically or hybrids of Indian-China varieties (**Figure 3**; **Supplementary Table S5**). In addition, 130 and 100 cultivars had a mixed pedigree belonging to P1 and P2, respectively (**Figure 3**; **Supplementary Table S5**). The recent introgression from P1 into P2 and vice versa had been so intensive that some of different populations did not reflect their geographical distributions. These results were consistent with previous studies (Warschefsky and Wettberg, 2019; Wang et al., 2020). Warschefsky and Wettberg (2019) identified two gene banks for cultivated mango accessions and further identified them as Indian or Southeast Asian types, but there was no significant bottleneck between the two types of gene banks. Their results indicated that mangoes had a more complicated domestication history than previously speculated. Wang et al. (2020) reported that the genome resequencing revealed two different groups of mango germplasms; commercial varieties were clustered together with Indian germplasms, which showed an allelic admixture. Southeast Asian native varieties were in the second group. Chinese varieties had formed different branches, and some varieties showed admixture in the genome. In the present study, 25 and 29 accessions kept their homogeneous genetic background in P1 and P2, respectively, and most of accessions (80.99%) were of mixed ancestry. This mixture may be the result of human hybridization, domestication, and selection, spread through human migration or trade, which had a great impact on the diversity of population structure.

### Insight into mango breeding history and breeding strategy

Understanding the source of cultivars is very important for clarifying the breeding history of cultivars, assessing genetic diversity and promoting breeding strategies. However, the spread of germplasm resources and the breeding of new varieties may be very complicated and difficult to understand, because many germplasm resources spread and the breeding process of variety previously had no detailed historical records (Nishio et al., 2014).

The mango breeding program in China has gone through two stages: the first stage was that of seedling breeding, in which a large number of original varieties were selected from seedling of Indian or Southeast Asian type varieties (P1 or P2); the second one was that of cross breeding, in which the breeding of artificial cross between P1 and P2 had produced many excellent varieties, which had been widely promoted in China (Luo et al., 2010; Huang et al., 2013; 2012).

Molecular genetics studies on the spread process of cultivars had been carried out in previous studies (BJaS, 1997; Olano et al., 2005). Parentage studies have shown that sexual cross breeding has played an important role in the emergence of new varieties in the past, and these excellent varieties had been adopted and spread by vegetative propagation (Bowers, 1999; Crespan et al., 2008). As expected, in the current research, the genetic relationship between seedlings selected cultivars or offspring of hybrid and their parent cultivars was closely related according to the dendrogram (**Figure 6**; **Supplementary Table S1**).

At the beginning of the twentieth century, the introduction of mango germplasm resources and the breeding programs of new varieties were strengthened in USA, especially in South Florida, where many of the most important commercial cultivars were released; many of those were still cultivated in major tropical regions around the world. The elite characteristics of these cultivars and their success all over the world had made South Florida known as the second center of domestication (Knight and Schnell, 1994). There were 25 of 39 America accessions grouped into cluster I-1 (**Supplementary Table S1**; **Figure 6**), of which 13 accessions derived from seedlings of ‘Haden’ or derived from ‘Haden’ hybrids; the remaining accessions came from hybrids of early mango introduction in America such as ‘Mulgoba,’ ‘Turpentine,’ ‘Sandesina,’ and ‘Bombay’. Population structure analysis showed that these American mango varieties had a mixed and higher proportion ancestry of Indian mango type (**Supplementary Table S1**); these results indicated that the USA varieties were more closely related to Indian varieties than to Southeast Asian varieties, in agreement with previous analysis using 25 microsatellite loci (BJaS, 1997). Given that these varieties had a much broader genetic base and were the ancestors of the world’s most popular commercial varieties (Knight and Schnell, 1994; Olano et al., 2005; Singh et al., 2016), these commercial mango varieties possess very unique genes involved with a wide range of adaptability and production stability in growing regions around the world (Mukherjee, 1997). For this reason, we may infer that the American varieties in cluster I-1 constitute a heterotic group (H1). Exceptionally, accession ‘Edward’ was grouped into cluster II (Southeast Asian type); the result might represent human disturbance such as cross between cluster I and cluster II or production and movement of seeds; as expected, pedigree results indicated that this accession derived from ‘Haden’ × ‘Carabao’, a hybrid between Indian types (P1) and the Southeast Asian types (P2) selected under south Florida conditions; population structure analysis showed that ‘Edward’ has a mixed ancestry, 54.6% Southeast Asian type ancestry and 45.4% Indian type ancestry. The results indicated that ‘Edward’ was on the borderline of the genetic population between P1 and P2.

During the first stage (seedling breeding) of the mango breeding program in China, a large number of original varieties were selected from seedlings of Indian or Southeast Asian type varieties (P1 or P2); however, only a small number of selected varieties have been widely planted, of which 25 of 39 Thailand mango accessions were grouped into cluster II-1; a large number (N = 21) of new mango varieties had been selected using seedling breeding from Thai mango germplasm resources which were released and promoted in China, such as varieties ‘Guire No. 3,’ ‘Guire No. 7,’ ‘Guire No. 10,’ ‘yuanjiang_ivory,’ ‘haibao 1,’ ‘guire 71,’ ‘hongxiangya,’ ‘Jinsui,’ ‘Wugongci Mango,’ ‘Guire 11,’ and ‘Hwagnyu (503)’ (**Supplementary Table S1**). Furthermore, Thailand mango accessions contain unique genetic diversity compared with other mango accessions (Warschefsky and Wettberg, 2019); from this perspective, Thailand mango germplasms may constitute another heterotic group (H2).

Varieties cultivated widely were facing common problems of degradation with longer cultivation, poorer resistance to pests and diseases, poor fruit quality, and sensitivity to abiotic stress (Schulze-Kaysers et al., 2015). It is important to expand the genetic base by introducing more exotic germplasm resources. Over the last decades, Chinese breeders had successfully bred several superior commercial mango varieties through cross breeding (**Supplementary Table S1**). The present results showed that mango accessions might be divided into two groups, which was consistent with the two proposed centers of domestication, and allelic admixture was observed in the genomes of commercial varieties. Interestingly, several important commercial mango varieties have been selected through cross breeding between American (H1) and Thailand mango accessions (H2) in China such as ‘Jinhuang,’ ‘Yiwen,’ ‘Guixiang,’ and ‘Guifei’ which were evenly distributed in the phylogenetic tree (**Figure 6**; **Supplementary Table S1**); of those, ‘Guifei’ was the most popular commercial variety in Taiwan, Hainan, and Guangxi provinces, and ‘Jinhuang’ was the most popular commercial variety in Taiwan, Yunnan, and Sichuan provinces in China. These results indicated that new varieties through cross breeding combine the advantages of two heterosis groups and have elevated levels of diversity.

## Conclusions

The exchange between the two groups (P1 and P2) elevated levels of diversity of mango accessions; the results of their genetic diversity and pedigree indicated the blood relationship of the main commercial varieties in China. Our results indicated that the proposed heterotic group existed in P1 and P2, respectively; hybridization occurred widely between P1 and P2, and most of accessions (80.99%) were of mixed ancestry, perhaps including multiple hybridization events and regional selection. The present work may have direct implications of a new strategy for mango breeding and germplasm management, which merits further investigation.

## Data availability statement

The datasets presented in this study can be found in online repositories. The names of the repository/repositories and accession number(s) can be found below: Bioproject accession number: PRJNA1037345.

## Author contributions

QL: Funding acquisition, Writing – original draft. HP: Writing – original draft, Data curation. XH: Data curation, Formal analysis, Writing – original draft. SW: Conceptualization, Data curation, Writing – original draft. YH: Data curation, Formal analysis, Investigation, Writing – original draft. HX: Writing – original draft. GX: Conceptualization, Data curation, Formal analysis, Investigation, Writing – original draft. RY: Writing – original draft. DL: Data curation, Investigation, Resources, Writing – original draft. ZY: Supervision, Writing – review & editing.
